# Developing a model of family focused practice with consumers, families, practitioners and managers: a community based participatory research approach

**DOI:** 10.1186/s12913-018-2844-0

**Published:** 2018-01-30

**Authors:** Andrea Reupert, Bernadette Ward, Francis McCormick, Cathy Ward, Susan Waller, Susan Kidd

**Affiliations:** 1 Faculty of Education, Krongold, Monash University, Clayton, VIC 3800 Australia; 2 School of Rural Health, Monash University, PO Box 666, Bendigo, VIC 3552 Australia; 3 Psychiatric Services, Bendigo Health, PO Box 126, Bendigo, VIC 3552 Australia; 4 Murray City Coast Country GP Training, Bendigo, VIC 3550 Australia; 5 Monash Rural Health, Monash University, PO Box 397, Moe, VIC 3825 Australia

**Keywords:** Mental health, Clinical practice/guidelines/resource use/qualitative research

## Abstract

**Background:**

While governments are urging adult mental health services to support consumers in the context of their family, there is little information about what family focused practice is, nor how it might be enacted.

**Methods:**

Informed by the principles of Community Based Participatory Research, workshops were held in three rural Australian communities in 2015 to discuss the meaning of family focused practice and how such practices might be promoted.

**Results:**

Participants described the need to raise community awareness about mental illness and provide practical support to the family. Participants emphasized the importance of practitioners genuinely communicating with consumers and their families about mental illness and the need for collaborative care and treatment planning. They also highlighted the challenges of living in rural places and posed some solutions.

**Conclusion:**

On the basis of the results and previous literature, we developed a model of family focused practice that outlined various stakeholders and their enactments. The model has the potential to inform policy, professional development and practice guidelines.

## Background

The mental health of an individual will inevitably impact on the wellbeing of his or family. The reciprocity between family members infers that an individual’s mental illness impacts other family members’ wellbeing, and in turn, family functioning may impact the individual with the illness. Accordingly, adult mental health services need to consider their clients within the context of family. This paper aims to develop a model of Family Focused Practice (FFP) and identify strategies that services might employ to promote a family focused approach within adult mental health services. FFP broadens the unit of care provision from a narrow focus on the mental health consumer to the wider family system [1].

Increasingly, governments and service providers across Europe, North America and Australia are promoting a family centred model of practice when working with consumers with mental health concerns. For example, the Canadian Rising to the Challenge [2] acknowledges that family members can foster support for recovery and well-being for those with a mental illness. Similarly, in Australia the 2014 Victorian Mental Health Act states that “children, young persons and other dependents of persons receiving mental health services should have their needs, wellbeing and safety recognized and protected” and should be “involved in decisions”. However, what FFP means in practice and how it might be embedded within services is unclear.

Rather than use the terms carer/caregiver, which may convey notions of dependency [3], the term ‘family’ is employed in this paper to include partners, parents, children, siblings, friends or other people who provide substantial support to an individual with a mental illness or have mutually interdependent relationships. We take a broad approach to the definition of family, which may not include one’s biological family, but instead consists of members who share a common purpose, set of conventions and customs [3].

Historically, FFP was commonly framed in relation to paediatric services where the presenting client was a child with a physical disability [4]. Here FFP denotes a collaborative approach when working alongside the child’s parents. In adult mental health services, Eassom et al. [3] suggest that family approaches might be considered on a continuum from relatively “basic functions” to specialized interventions such as Family Interventions (FIs). FIs are designed to address problematic family dynamics involving critical and emotionally over-involved attitudes that relatives have toward a family member with a disorder [5]. While FIs vary in range, depth and focus, they typically involve formal approaches (for example, the use of a manual) that aim to improve the emotional atmosphere of the family [6]. Rather than a prescriptive intervention, the focus in this study are practitioners’ daily, routine practices, embedded into treatment that may be employed to acknowledge and support clients and their families.

Notwithstanding the lack of a FFP framework, there are two main ways that a consumer’s family is discussed and portrayed in the mental health literature. First, FFP may relate to the manner in which services address the needs of family members who are caring for, or living with someone who has a mental illness [7] in order to improve their experience of caring and reduce the associated burden [8]. Instrumental, emotional and social support might be offered, in addition to psychoeducation [1]. Addressing family needs may lead to a reduction in subjective burden of care, increased levels of self-care and emotional role functioning [9]. Second, FFP may incorporate the contributions that family members make to the care of their relative. In the past, some paradigms and professional groups considered families to be the cause of a person’s mental illness [10]. Increasingly however family members are self-initiating or invited by providers to be actively involved in the care of their relative. Family members, typically parents, play different roles in an individual’s treatment including “offering hope, encouragement and opportunities” and providing “a steadfast belief in participants’ ability to recovery” [11]. They may also provide practical support such as monitoring medications, offering housing and assuming household chores [11]. Services might promote family support by encouraging the family to participate in the development and delivery of a treatment plan [12].

While noting the proliferation of policies in this area, Dempsey and Keen [13] suggest that in reference to a conceptual framework, “the family focused field can best be described as being in an adolescent phase of development”. FFP is often promoted as a best practice model of delivery [14] but there are numerous barriers to its implementation. One barrier is having a shared understanding as to what FFP involves; Foster, et al. [1] argue that it is difficult for services to practice FFP if there is no consistent, clear framework.

The aim of this study was to inform the development of a FFP model. We sought to elicit from consumers, family members, mental health practitioners and managers what FFP is and the specific ways FFP might be promoted, within their own communities. Such information might be used to inform policy, professional development and practice guidelines, especially within rural communities.

## Methods

### Design

The design and reporting of this project was informed by the principles of Community Based Participatory Research (CBPR) defined as “systematic inquiry, with the collaboration of those affected by the issue being studied, for purpose of taking action or effecting change” [15]. The CBPR approach recognises consumers, family members and practitioners as producers of knowledge and not just research project participants [16]. CBPR utilises innovative methods to resolve a problem and recognises the importance of stakeholders’ capacity to analyze knowledge while articulating their own needs [15]. In the present study, this co-learning process was framed within a mutual exchange of expertise amongst those that live with mental illness, family members, those who work in the field and the project team who facilitated the process. A key strength was the integration of researchers’ theoretical and methodological expertise with non-academic participants’ experience and knowledge, to respond to issues in a comprehensive and coordinated manner.

### Context

The study was conducted in rural Australia, across the states of Victoria and New South Wales where practitioners service a population of 230,000 across 50,000 km^2^ [17]. In both states, acute and community mental health services are predominantly provided by government funded services including specialist and/or general hospital, community and medical practitioners. Rural mental health presents particular challenges. Compared to urban areas, accessing mental and other health care services is more difficult and rural populations have worse population mental health outcomes [18]. Understanding and addressing the particular mental health needs of rural areas requires, among other things, access to the voice of stakeholders, specific to these contexts. As per Israel et al. [19] the project team recognised the community as a social entity rather than merely a setting and sought to engage in what community members considered the approaches that would best meet their context.

### Procedure

A reference group, consisting of consumers, family members and practitioners from community and mental health sectors initiated the design and funded the Partners in Recovery project group, consisting of researchers and practitioners. Subsequently, a member of the project group conducted individual interviews with consumers, family members and practitioners to discuss their experiences of FFP and identify strategies for how the subsequent community workshops might be promoted and facilitated. In this part of the project, there were interviews with 11 mental health practitioners, 12 consumers, and 9 family members (total *n* = 32). Interviews were conducted by three members of the project team, two with extensive experience working in mental health and all with experience in conducting interviews. Transcripts were then analyzed by all six members of the project team (three researchers, three practitioners). A conventional content analytic approach was employed whereby each member of the team independently read through each transcript several times, and derived codes by highlighting specific sections of text [20]. Then, working together as a group, the team identified labels for codes and subsequent categories based on the relationships between these different codes. The team subsequently employed these emergent categories to group codes into meaningful clusters that were used in the subsequent workshops. These different activities promoted participation, trust and respect among different stakeholders.

The design of the workshops was informed by de-identified data collected during these preliminary interviews. A thematic summary of interview participants’ experiences of FFP was presented at the start of each workshop. Participants were invited to consider whether anything was missing but, arguably more importantly, to consider what FFP meant, and what actions were needed for FFP to be successfully translated into their community.

Each workshop was two hours long, and conducted in a central, public venue, with lunch provided. Participants were invited to sit in allocated seating, in small groups around a table. Pre-determined groups and numbers ensured there was an equal distribution of practitioners, managers, consumers and family members at each table. It also meant practitioners and managers from the same organisation did not sit together, nor did consumers and their family member (unless they requested to do so).

The overall structure of the workshops were as follows:

1. Introduction and workshop objectives
2. Outline concepts of being a “safe space”:
  - Importance of listening to each other
  - The need to work together on shared goals
  - Not a blaming or shaming exercise for any particular agency
  - Each participant to take some responsibility for following through on any action plans generated
3. Ice breaker to get to know each other outside of the “label” of consumer, family member, practitioner or manager
4. Background information using selected interview excerpts collected previously
5. Each table discussed and recorded on large sheets of paper:
  - what FFP is; and
  - how to promote FFP in their particular community.

Project team members facilitated each table. Each table then reported back to the larger group.

6. A larger group discussion ensued, whereby participants were invited to reflect on all the information presented.

A member of the research team took field notes on each of the workshops, that included both descriptive information (e.g., what happened when, who said what) and reflective information (reflections about the interactions and what was said/not said). These were then discussed with the research team and together with the written information generated by each table, were analyzed as outlined below.

### Participants

Consumers, family members, mental health practitioners and managers were invited to attend one of three workshops in different towns. Purposive sampling [21] was used to recruit practitioners and managers who were (i) from relevant organisations and (ii) had worked with consumers and family members.

Convenience and snowball sampling [21] was used to identify consumers with persistent mental health concerns as well as family members. Flyers, community radio, noticeboards and Facebook pages were used to promote the workshop and recruit consumers and family members residing within 50 km of each town. The selection criteria for consumers was that they needed to be sufficiently well to participate. Formal screening of participants was not conducted. Instead, one of the research team discussed the nature of the project with potential participants, and ensured they understood what was expected of their participation before providing details about the venue, date and so on. Both family members and consumers were clearly informed that practitioners would be in attendance and that they may have worked with them previously. It was not a requirement that consumer’s relatives participated, nor for the consumer of relatives to attend.

### Data collection, analysis and the interpretation processes

The CBPR approach calls for new methods of data collection, and the question of "appropriateness of the method to the participants" is particularly relevant here [22]. As early as 1967, Glaser and Strauss [23] stressed the need to conduct data analysis in groups not limited to researchers only. This particularly applies to CBPR because it requires that various perspectives flow from data collection into the process of data interpretation [22]. As outlined above, we designed, collected, presented, discussed and interpreted data iteratively as the workshops progressed and this encouraged ownership of the research process; a central component of a CBPR approach. This ultimately ensured the overall multi-perspectivity and multivocality in the representation of the results [24].

Within this participatory approach, we employed a qualitative, interpretative framework, to guide analysis. The iterative nature of qualitative research that attempts to make meaning from different viewpoints was considered to be well aligned with the participatory approach framing this project. As per Cornish et al. [25] by collaborative data analysis we refer to processes in which there is joint focus and dialogue among different stakeholders regarding a shared body of data, to produce an agreed interpretation. This approach is espoused by Smith [26], an exponent of interpretative phenomenological analysis, who argues that analyzes should reflect both the speaker’s voice (the primary data) along with a critical engagement of the text, treating the speaker’s voice as a result of social or psychological processes which call for an explanation. The careful balancing between what participants had to say at different points of the research process, along with an active interpretation of these data within the context of each community, was managed primarily by the project group though there were feedback loops at different points to maintain authenticity including, for example, the use of peer debriefing in the data analysis.

Data consisted of the written discussion sheets generated from the various tables along with the field notes taken from the larger group discussion by a member of the project group. As per the earlier stage of this research, a conventional analytic approach was employed to analyze all the workshop data [20]. Content analysis is the creation of codes generated from the data themselves and are as close to participants’ own language as possible, using focused, line-by-line coding to seek notable categories in the raw data [20]. In the first instance, two researchers conducted the analysis, both with extensive experience in qualitative research analysis, with one also having extensive experience as a practitioner with families living with mental illness. The themes identified were then shared and discussed with the project group, consisting of three researchers and three practitioners, to reach the final themes presented here. There was no distinction made between the views of the different workshop participants. While this might be considered a limitation, it could be argued that the results provide a form of co-produced consensual overview of FFP. After the three workshops, data saturation was reached as it was decided by the project team that collecting more data would not lead to more information.

### Ethics

Approval to conduct this research was obtained from the relevant research ethic committees. Participants were provided with an explanatory statement and gave written consent prior to workshop attendance. A detailed description of the workshop format was made clear. In recognition of the time and costs associated with attending, consumers and family members were provided with a retail voucher to the value of $70.

## Results

Forty-six people participated in the workshops. Most attendees were female and attendance was highest at workshop one (which was the largest of the three towns) (see Table 1).

**Table 1:** Workshop participants

N	
*Workshop one*	24
• Gender	
○ Male	3
○ Female	21
• Consumer	4
• Family member	12
• Mental health practitioners	1
• Mental health manager	7
*Workshop two*	14
• Gender	
○ Male	5
○ Female	9
• Consumer	3
• Family member	6
• Mental health practitioners	2
• Mental health manager	3
*Workshop three*	8
• Gender	
○ Male	–
○ Female	8
• Consumer	3
• Family member	2
• Mental health practitioners	3
• Mental health manager	–
Total number of	
• Consumers	10
• Family members	20
• Mental health practitioners	6
• Mental health managers	10
Total overall participants	46

There was much interest in attending the workshops, especially from family members. Of the consumers/family members who expressed interest in attending the workshops, 30 attended. At one workshop more family members wanted to attend (*n* = 20) than places available to them (*n* = 12). Common themes for understanding and promoting FFP were identified, as outlined below.

### Awareness raising and education

Workshop participants highlighted a need for general awareness raising with “the first thing that is needed is education for everyone about mental health”. Some suggested that the information delivered needed to be at an age appropriate level with the literacy level at grade 5/6 (age 10–13 years). Such information needed to be “out in the wider country” with “notices in the (local) supermarket” and covering “all stages of the (recovery) journey” including information about the chronic nature of the mental illness, where some people may not be ill all the time. Participants were clear that such educational initiatives would address stigma and prejudice and promote understanding of the difficulties faced by consumers and families. There was also some reporting of specific groups that need to be targeted, including “Parole board, police and teachers”.

Additionally, participants highlighted the type of information that family members and consumers wanted to know at different times including “on diagnosis or at initial presentation…[the] person and family to be offered mental health first aid training”. Another suggestion was for local information packs, which included generic psychoeducation on mental illnesses and information about services and procedures. There was a need for family members to know what was happening to their relative when he or she was admitted into hospital and while being treated over the longer term. Another suggestion was to “make small local hospitals available – [so we know] which inpatient service you can use and when”. Participants wanted to have services available in their local community so they did not have to travel, especially for emergency services.

### Practical support to the family

A constant theme across workshops was the need for practical support for family members. Access to transport featured commonly, especially when visiting their relative in hospital, “It would be great if there was transport for carers to [large rural town] when the person is inpatient there” but also for travelling to other services for themselves and for their relative. One suggestion was for practitioners to travel to families, rather than the other way around. One group pointed out that there is an “expectation that workers travel for professional development and team meetings BUT this expectation isn’t built into client care”. Another theme was the provision of respite for relatives, from caring for their family member. In particular, after hours care, when things were not going well for the consumer was a topical issue across workshops that was considered important for both the consumer and his or her family.

### Genuine communication

The common problem reported across the groups was the requirement to “tell our story again and again”. Accordingly, there were suggestions for agencies to include the consumer in discussions around treatment plans and for practitioners to have regular meetings with the consumer and his or her family, both together and separately. Similarly, participants were clear that there needed to be better communication between services especially between medical and mental health staff e.g., “better communication/support between consumers, family members and pharmacists with regards to management of medications”. Participants emphasized the need for inpatient staff to provide information to families regarding how their relative’s progress, when they might be discharged and how they might support them once they left. One group suggested that this communication might include a video link.

Relatedly, concerns were raised about confidentiality and the tensions around releasing information to family members. When discussing whether families should be updated about their ill relative, one manager publically and assertively declared that ‘we [the service sector] tend to hide behind confidentiality” and therefore did not share information with family members. One group recorded that it “should be automatic that family is included” and in the group discussion stated that there should be “opt out of sharing information, not opt in… [so that] sharing is the default”. Similarly, other comments included, “Confidentiality – needs to be worked with” and “[we need] creative ways of working with privacy legislation”. Some suggested that general psychoeducational information could be released to the family without violating confidentiality. Consumers indicated that they were happy for their information to be shared as “it made it easier for everyone, and I don’t have to try to explain what my psych said to me”. However, consumers still wanted to be asked for their permission to release information. Participants were clear however that family members had rights and could not be expected to support their relative without at least some knowledge of what was happening to him or her.

### Collaborative care and treatment planning

There were repeated requests for families to be involved in planning the treatment of their relative. One suggestion was that there could be a nominated case manager who would “coordinate care, communication between family, caregivers, health practitioners to facilitate the process of the ‘nuts and bolts’ of care”. Also, participants emphasized the need for crisis planning around the relative’s illness:

Being assisted to plan what we want to happen when [relative’s name] is unwell, before it happens.

[The] family needs strategies to help with acute times/episodes.

Discharge was another time which needed to be carefully planned; “Discharge planning [needed to occur] from the start” that involved “liaising with everyone”. Other comments regarding the discharge process included:

On discharge, it would be helpful to give information to consumers/carers [about other services in the local community].

[a discharge] email is not enough – [the] discharge plan [needs to] involve all parties.

[after discharge there needs to be] a softer entry point to post-acute services rather than just giving a service name and phone number.

### The rural context when working with families

The rural context of these communities impacted the nature of family orientated practices. Concerns were raised by participants that “we get forgotten because we live in the country…” Participants suggested that services needed to “recruit country people for country [mental health] positions”. Additionally, telehealth and Skype was identified as two strategies that might be usefully employed to support family members and consumers though it was also noted that there needed to be choice as “some people [would] choose to be there in person”. Another suggestion was for services to provide a “video link to “visit” with the family member” or through “Skype and Facebook”.

## Discussion

This study sought to provide a framework for FFP, grounded in the lived experiences of consumers, family members, practitioners and managers. Corroborating the intertwined nature of family relationships identified by others [27], the study confirms the basic principle that families are important when working with those with mental health issues.

FFP involved supporting the family’s own needs (for example, through education, respite and transport), which resonates with previous literature [7]. Additionally, FFP was described as a process in which family members and practitioners might collaboratively plan for, and work with, the consumer on his or her treatment plan, and treatment progress and care including crisis planning. Reupert et al. [28] propose developing family care plans that incorporate both crisis and care components; the former involves identifying warning signs and having emergency contacts on hand, while the latter includes a treatment plan for each member of the family. Participants in this study identified discharge as a critical time for families and services to work together, to actively discuss the types of services that they and their relative might work with, on an ongoing basis. Various critical time points were seen as opportune times for inviting family involvement and these need to be capitalised by mental health agencies.

Workshop participants noted that FFP involved genuine communication between various stakeholders, regarding what was happening for the consumer as well as treatment decisions and progress. Likewise, others have highlighted the importance of consumers maintaining contact with their families while in hospital [29]. These findings speak not only to providing accurate and timely health care information but also a practitioner’s interpersonal sensitivity to the feelings and concerns of the family, communication efforts that seek to build partnerships, and creating opportunities for families to share their perspectives and concerns. The importance of interagency communication and collaboration that was highlighted in this study also resonates with previous literature [30].

While confidentiality is often highlighted as a barrier for working with families [30], it is interesting that one manager suggested that services “hide behind confidentiality” while another participant suggested that services need to be “creative” within this legislation. While there was broad agreement that families needed to be informed, obtaining consumer permission was still critical. The tension around whether to, and how to release consumers’ treatment information to their family has been discussed elsewhere [29, 31]. Marsh and Johnson [29] suggest that if the release form is presented at the right time (when the consumer is sufficiently well to provide informed consent) and when explained by the practitioner as a means of enhancing treatment, most consumers are willing to authorise the release of relevant information to their families, a strategy that would appear to be aligned with the strategies noted by workshop participants here. As outlined by participants, general psychoeducational information about symptoms, medication and treatment can be shared with families, without concerns related to confidentiality.

Another point raised in workshop discussions was the need for general education about mental illness as means of promoting FFP in the community. This need arises from consumers’ and families’ experience of stigma and prejudice that may lead to marginalisation and ostracism [32]. Education might be provided that promotes the view that mental health problems are a part of our “shared humanity”, an illness that anyone can develop [33]. Public education around the high prevalence of mental illness may also reduce the image of illness as a problem of “others” and hopefully the associated stigma [34].

The rural context influenced the way in which FFP was delivered. Strategies for recruiting practitioners who grew up in rural areas has been mooted elsewhere as a key strategy for maintaining a rural health workforce [35] and one that was appealing to participants. Telehealth technologies were also proposed as ways of overcoming some of the problems typically associated with providing mental health services in rural areas. While telehealth can be as effective for face to face consultations [36] these modes might also be utilised for maintaining contact between family members, between the family and the practitioner and to facilitate ways for families to participate in collaborative treatment meetings.

**Fig. 1:**
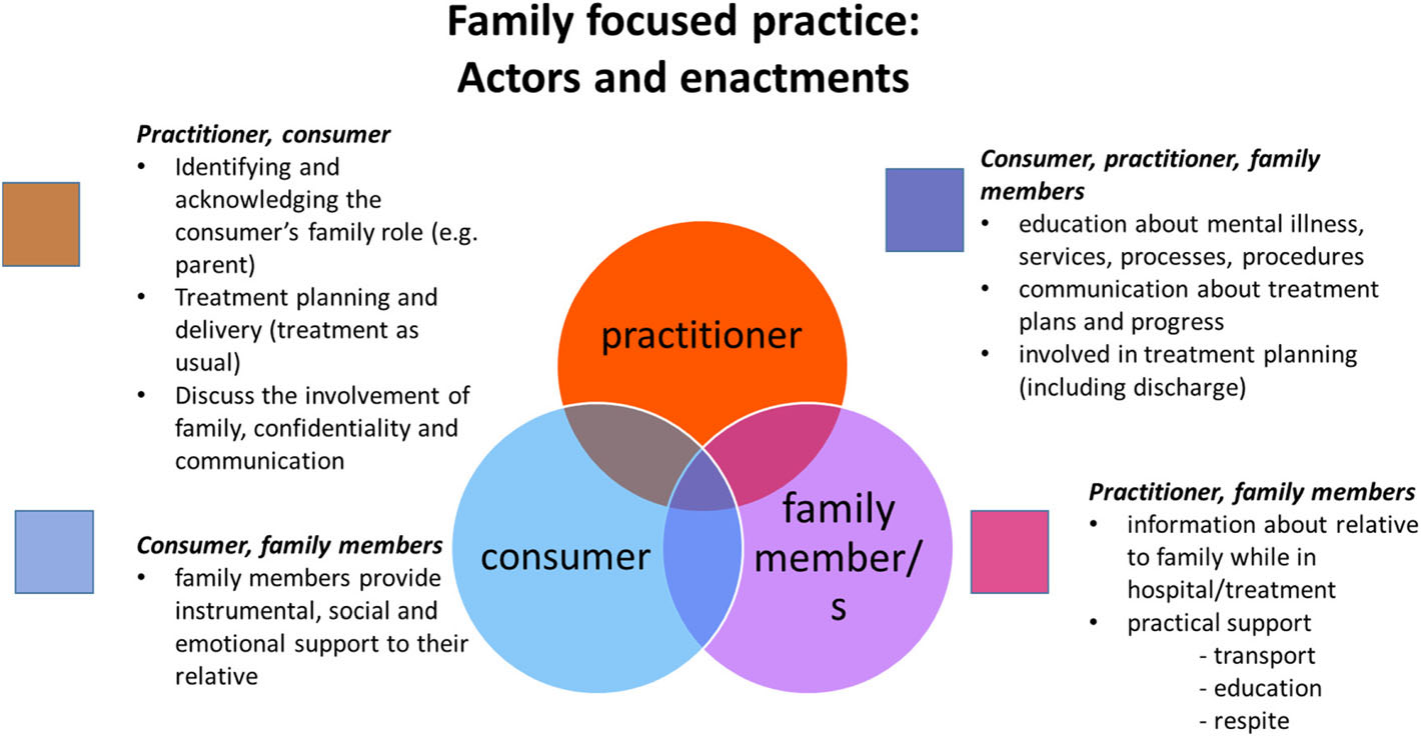
Family Focused Practice: Actors and enactments

One of the aims of this paper was to develop a model of FFP that might be incorporated into practice standards and professional development activities. Drawn from the results and our reading of existing literature, FFP can be conceptualised through specific activities or enactments (e.g., communication) and particular ‘actors’ (namely, the family, consumer, and practitioner) (see Fig. 1).

First, FFP includes the instrumental, social and emotional support the family provides to their relative. This component of the model acknowledges the critical role that families play in providing care. Another sphere involves the practitioner and the consumer. This sphere incorporates treatment as usual (assessment, treatment planning and delivery and discharge). Embedded within treatment is the practitioner having a discussion with the consumer about the involvement of family e.g., how the consumer defines family and to what extent and how various family members might be involved and when. Inherent in these discussions are arrangements pertaining to confidentiality and communication more generally.

FFP also involves particular enactments that occur between the practitioner and the family. These enactments include the practitioner addressing the family’s emotional and practical needs for respite and transport (or organises for another agency to do so). This sphere includes a practitioner providing information to the family about their relative, an orientation to services, psychoeducation about their relative’s illness and ways to support the consumer and manage crisis times is included here. Family members have much to offer the practitioner also, who may be consulted and involved in providing information for assessment and when developing treatment plans. Organisational support to embed such enactments include having FFP as one of the selection criteria for new staff and incorporated into position descriptions of clinical staff. Adequate time for this work and professional development in this area, including supervision, is also critical.

The final sphere involves all three stakeholders, consumer, family and practitioner, where all three share information and work collaboratively on assessment and treatment plans. The ideal is to recognise the strengths and skills each stakeholder brings and establish mutually agreed treatment goals where the roles of each are clearly defined. While practitioners might educate the family and consumer about mental illness or service procedures, the family and consumer can educate the practitioner about their experiences of the mental illness and services.

It is important to note the broader community and organisational context, in which all three stakeholders and their various enactments are situated. The relationships that organisations have with other organisations and the interprofessional exchanges that are needed for FFP is not featured in this model but do provide further contextual detail.

This study is limited to the perspectives of workshop attendees who do not necessarily represent the views of all rural people. Participants may have been reluctant to disclose information in a group format, given the mix of practitioners, managers and consumers. There is a potential for group bias, where everyone merely agrees with each other. Children whose parents have a mental illness are critical family members [37] and future research needs to incorporate young people’s perspectives. Ideally, it would be useful to facilitate further workshops similar to those conducted here, with family members, consumers and practitioners. Discussions could be facilitated to consider different components of the model with the various stakeholders. For example, consumers and family members might be invited to consider whether the enactments listed here encapsulate their relationship, and what more might be needed and/or changed. The role of context in terms of organisational culture could also be explored in these discussions, regarding the enactments that might vary in different settings such as for example, inpatient versus rehabilitation services.

Nonetheless, the model presented here provides some guidance as to what FFP is, and how it might be enacted. Practical implications arising from this study and the resulting model are to (i) ensure intake procedures identify family members and their current and possible future involvement, (ii) provide training and supervision to practitioners that promotes skills in engagement and communication with consumers and family members for example, how practitioners might discuss the involvement of family with consumers and (iii) identify pathways of care for family members in terms of psychoeducation, respite and transport. Organisations need to (i) recruit staff who have a genuine willingness to engage and listen to families, (ii) ensure that working in partnership with families and consumers is a core feature of practitioners’ positon descriptions and (iii) clarify partnership processes and goals from the outset with families as well as other agencies. Finally, the input of families and consumers should be encouraged regarding the nature of services offered, something this study has attempted to emulate. Implementing practice change can be challenging and time consuming [31] but having a model, such as that generated here, may provide, in part at least, impetus for FFP leadership.

## Conclusion

Mental illness impacts on more than the individual; inevitably other family members are impacted by, and will respond to the illness, in varied ways. Embedding FFP into standard routine care has the potential to reduce family distress, improve family functioning and deliver substantial benefits for the consumer. There is a paucity of practice models that demonstrate how FFP might become a standard part of routine care. Elicited and synthesized from the views of consumers, family members, practitioners and managers, along with previous research, a model of FFP was developed with specific actors or stakeholders and enactments. The model needs to be further developed with families, consumers, practitioners and managers.

## Abbreviations

CBPR: Community Based Participatory Research
FFP: Family focused practice
